# Activation of the STING pathway potentiates the antitumor efficacy of doxorubicin in soft-tissue sarcoma

**DOI:** 10.3389/fonc.2025.1634503

**Published:** 2025-12-12

**Authors:** Wonyoung Choi, Gi Yeon Lee, Sun-Young Kong

**Affiliations:** 1 Center for Clinical Trials, National Cancer Center, Goyang, Republic of Korea; 2 Division of Cancer Biology, National Cancer Center, Goyang, Republic of Korea; 3 Division of Rare and Refractory Cancer, National Cancer Center, Goyang, Republic of Korea; 4 Department of Cancer Biomedical Science, National Cancer Center Graduate School of Cancer Science and Policy, Goyang, Republic of Korea; 5 Department of Laboratory Medicine, National Cancer Center, Goyang, Republic of Korea

**Keywords:** soft-tissue sarcoma, STING pathway, immunostimulation, chemotherapy, doxorubicin

## Abstract

**Background:**

Systemic treatment of soft-tissue sarcoma (STS) relies on cytotoxic chemotherapy, with doxorubicin being the key therapeutic agent. However, immune activation is required for optimal antitumor effects of doxorubicin. This study investigated whether activation of the STING pathway enhances doxorubicin’s antitumor effect in STS.

**Methods:**

STS cell lines were treated with doxorubicin to evaluate the activation of STING pathway. Deletion of *Sting1* gene was employed to validate its role in mediating doxorubicin’s effects. In a syngeneic mouse model of STS, doxorubicin was administered alone or in combination with a STING agonist ADU-S100. Tumor-infiltrating CD45^+^ cells were magnetically sorted for RNA sequencing to identify genes and pathways linked to STING activation. The upregulated genes were analyzed for their association with survival in the Cancer Genome Atlas Sarcoma (TCGA-SARC) patient cohort.

**Results:**

Doxorubicin induced cytosolic DNA leakage in STS cell lines, triggering the activation of STING pathway. Deletion of *Sting1* attenuated doxorubicin-induced upregulation of proinflammatory cytokines in cells. In the syngeneic mouse model of STS, doxorubicin suppressed tumor growth, an effect significantly enhanced by coadministration of ADU-S100. RNA sequencing of tumor-infiltrating CD45^+^ cells revealed upregulation of immune pathways linked with STING signaling. In TCGA-SARC cohort, patients with higher expression of genes upregulated in the cells from STING-activated tumors exhibited improved survival, whereas those with lower expression showed poorer overall survival.

**Conclusion:**

Activation of STING pathway by ADU-S100 enhances the antitumor efficacy of doxorubicin in STS. Combining doxorubicin with STING agonists may be a promising therapeutic strategy worth exploring in future clinical trials.

## 1 Introduction

Soft-tissue sarcoma (STS) is a rare cancer, accounting for only 1% of all malignancies, with an annual incidence of 2.49 per 100,000 person-years in Korea and a global age-standardized incidence rate of 1.16 in every 100,000 people (1, 2). About 11.9% of patient with STS present with distant metastasis at the time of diagnosis (3), and up to 40% eventually develop metastatic disease (4). Despite the heterogeneity of STS, which encompasses over 100 distinct subtypes of mesenchymal tumors, systemic treatments for metastatic diseases are mostly selected without consideration of the subtype (5). Moreover, the scarcity of actionable oncogenic alterations and relatively low tumor mutational burden in STS limits the efficacy of targeted therapies and reduces the clinical benefits of immune checkpoint inhibitors (6, 7). Due to these challenges, cytotoxic chemotherapy remains the cornerstone of systemic treatment, with doxorubicin being the first-line drug-of-choice for STS (8, 9). Doxorubicin intercalates into DNA, interfering with the topoisomerase activity. However, recent findings have highlighted the pivotal role of the immune system in mediating its antitumor effects (10–12). As such, the activation of proinflammatory cascades and stimulation of interferon (IFN) signaling pathways may augment the antitumor activity of doxorubicin.

The stimulator of interferon genes (STING) pathway is a key innate immune mechanism that detects cytosolic DNA and activates downstream signaling, resulting in the production of type I IFN and other immunostimulatory cytokines (13). This pathway is regulated by cyclic GMP-AMP synthase (cGAS), which interacts with cytosolic DNA and generates the cyclic dinucleotide cGAMP. This secondary messenger binds to and activates STING located on the endoplasmic reticulum. Upon activation, STING translocates to the Golgi apparatus, initiating a signaling cascade that activates TANK-binding kinase 1 (TBK1) and subsequently the transcription factor IRF3. This leads to the production of type I IFNs and other proinflammatory cytokines, which are crucial for antiviral defense, cancer immunosurveillance, and immune-mediated inflammation (14). In this context, the STING pathway has emerged as a promising target for antitumor immunotherapy (15).

Clinical responses to doxorubicin remain limited in patients with metastatic STS, with an objective response rate of 14%–18% and a median progression-free survival of 4–6 months (4, 9). Given this significant unmet clinical need, we investigated whether costimulation of the STING pathway could enhance the antitumor effects of doxorubicin in STS. Toward this end, we utilized ADU-S100, a synthetic cyclic dinucleotide that activates the STING pathway and has been tested in clinical trials (16, 17). Our findings provide a proof-of-concept for the therapeutic potential of STING agonists in STS treatment.

## 2 Materials and methods

### 2.1 Cell culture

Mouse sarcoma cell lines CCRF S-180 II, and WEHI-164, and human sarcoma cell lines HT-1080 (RRID: CVCL_0317), were obtained from the Korean Cell Line Bank (KCLB, Seoul, Korea), and MES-SA (RRID: CVCL_1400) was obtained from the American Type Culture Collection (ATCC, Manassas, VA, USA). All cell lines were authenticated by short tandem repeat (STR) profiling within the past three years to confirm their identity. The STR profiling was performed by the Genomics Core Facility of National Cancer Center, and results were compared against reference profiles. Prior to experimental use, all cell lines were routinely tested and confirmed to be free of mycoplasma contamination using a PCR-based mycoplasma detection assay.

CCRF S-180 II cells were cultured in DMEM supplemented with 10% fetal bovine serum (FBS). WEHI-164 and HT-1080 cells were cultured in RPMI 1640 medium supplemented with 10% FBS. MES-SA cells were cultured in McCoy’s 5A medium. All culture media were supplemented with penicillin (100 U/ml), and streptomycin (100 mg/ml). Cell lines used in this study were all authenticated by DNA fingerprinting and were routinely tested for Mycoplasma.

### 2.2 Cell viability assay and determination of IC_50_ values

Cell viability following doxorubicin treatment was assessed using the CellTiter-Glo^®^ 2.0 Cell Viability Assay (Promega, Madison, WI, USA) according to the manufacturer’s instructions. Briefly, cells were seeded in 96-well plates (2 × 10³ cells per well) and allowed to adhere overnight. The next day, cells were treated with serial dilutions of doxorubicin for 48 hours. Following treatment, an equal volume of CellTiter-Glo reagent was added to each well, and luminescence was measured using a microplate reader (Infinite 200 Pro, Tecan, Switzerland). Relative cell viability was normalized to untreated controls. IC_50_ values were calculated by nonlinear regression analysis using a four-parameter logistic model in GraphPad Prism (version 8.3.1). All experiments were performed in six replicates and repeated independently at least three times.

### 2.3 Immunofluorescent staining

Cells were seeded onto 8-well Lab-Tek II chamber slide (Thermo Fisher Scientific, #154534) and allowed to adhere overnight. Doxorubicin of IC_50_ doses for each cell line or DMSO (vehicle) was applied for 48 hours. After treatment, cells were washed three times with cold PBS and fixed with cold methanol at -20°C for 10 minutes. Blocking was performed with 1% bovine serum albumin (BSA) in PBS for 1 hour. For γ-H2AX staining, cells were incubated with a primary antibody against γ-H2AX (Ser139) (Cell Signaling #2577, 1:800 dilution) at 4°C overnight. After washing twice with PBS, cells were incubated with a goat anti-rabbit Alexa Fluor 594 secondary antibody (Thermo Fisher Scientific, #A-11012, 1:500) for 1 hour at room temperature in the dark. For PicoGreen staining, Quant-iT PicoGreen dsDNA reagent (Thermo Fisher Scientific, 3μl/mL) was applied at 37°C for 1 hour. Nuclei were counterstained with Hoechst 33342 (1:500 dilution) for 10 minutes at room temperature, and fluorescent images were captured using a confocal microscope. Staining intensities were calculated using ImageJ software.

### 2.4 Gene expression analysis by RT-qPCR

RNA was isolated from cultured cells using TRIzol (Thermo Fisher Scientific), and complementary DNA was synthesized from 1μg of total RNA with ReverTra Ace^®^ qPCR RT Master Mix (Toyobo, # FSQ-201) following the manufacturer’s protocols. Real-time quantitative PCR was performed using LightCycler 96 Real-Time PCR system (Roche) and Power SYBR Green PCR Master Mix (Applied Biosystems). Gene expression levels were calculated using the ΔΔCt (Delta–Delta Ct) method, with GAPDH serving as the endogenous control.

The sequences of the primers used in this study are listed below (**Table 1**).

**Table 1:** The sequences of the primers used for RT-qPCR in this study.

*Ccl5*	Mouse	Forward	GCTGCTTTGCCTACCTCTCC
		Reverse	TCGAGTGACAAACACGACTGC
*Cxcl10*	Mouse	Forward	CCAAGTGCTGCCGTCATTTTC
		Reverse	GGCTCGCAGGGATGATTTCAA
*Ifnb1*	Mouse	Forward	CAGCTCCAAGAAAGGACGAAC
		Reverse	GGCAGTGTAACTCTTCTGCAT
*Gapdh*	Mouse	Forward	AGGTCGGTGTGAACGGATTTG
		Reverse	TGTAGACCATGTAGTTGAGGTCA
CCL5	Human	Forward	TGCCCACATCAAGGAGTATTT
		Reverse	CTTTCGGGTGACAAAGACG
CXCL10	Human	Forward	GGCCATCAAGAATTTACTGAAAGCA
		Reverse	TCTGTGTGGTCCATCCTTGGAA
IFNB1	Human	Forward	GCTTGGATTCCTACAAAGAAGCA
		Reverse	ATAGATGGTCAATGCGGCGTC
GAPDH	Human	Forward	GGAGCGAGATCCCTCCAAAAT
		Reverse	GGCTGTTGTCATACTTCTCATGG

### 2.5 CRISPR-Cas9 knockout

*Sting1*-deficient cells were generated using the CRISPR/Cas9 system (18). In brief, the sgRNA sequences were cloned into the LentiCRISPR v2 vector containing the *Streptococcus pyogenes* Cas9 nuclease gene. The sgRNA sequences were designed using a web-based tool (https://crispr.mit.edu) (**Table 2**). Lentivirus was prepared in HEK293FT cells by co-transfection of the LentiCRISPR v2 vector and the viral packaging vectors pLP1, pLP2, and pLP/VSVG. Viral supernatant was collected 48 hours after transfection. Target cells were transduced with polybrene (8 μg/ml) and selected with puromycin for 2–3 days, starting 48 hours after transduction. Knockout effect was confirmed by immunoblot analysis of whole cell protein extracts. The guide RNA sequences used in this study are listed below.

**Table 2:** The sequences of sgRNA used in this study.

*sgSting1*	Mouse	5’ – CGGCAGTTATTTCGAGACTC – 3’
*sgNTC*	Mouse	5’ – GCGAGGTATTCGGCTCCGCG – 3’

### 2.6 Immunoblot analysis

Cells were lysed with radio-immunoprecipitation buffer (50 mM Tris-Cl, pH 7.5, 150 mM NaCl, 0.1% SDS, 0.5% sodium deoxycholate and 1 mM EDTA) containing protease and phosphatase inhibitors (1 mM NaF, 1 mM Na3OV4, PMSF, 2 mg/ml leupeptin and pepstatin; all purchased from Sigma) for 30 min at 4°C. The protein concentration was quantified with a Pierce BCA protein assay kit (Thermo Scientific). Blots were incubated with primary antibodies at 4°C overnight. The following antibodies were used for immunoblots: anti-STING (Cell Signaling #13647, 1:1,000) and anti-GAPDH (Cell Signaling #2118, 1:1,000).

### 2.7 Syngeneic graft and drug treatment

All mouse experiments were performed under approval by the Institutional Animal Care and Use Committee (IACUC) of the National Cancer Center. A total of 3 × 10^6^ WEHI-164 cells were subcutaneously inoculated near the thigh of BALB/c mice. Tumor volume (0.5 × length × width^2^) was monitored regularly using a caliper. When the tumor volume reached 200mm^3^, mice were received weekly intratumoral injections of doxorubicin (MedChemExpress, HY-15142) at a dose of 5mg/kg body weight and/or twice-weekly intratumoral injections of 25μg of ADU-S100 (InvivoGen, tlrl-nacda2r-01).

### 2.8 Tumor dissection and sorting of CD45^+^ cells

Grafted tumors were harvested 7 days after drug treatment and dissociated using the Tumor Dissociation Kit (Miltenyi Biotec, #130-096-730). The dissociated tumor tissue was stained with anti-mouse CD45 microbeads (Mitenyi Biotech, #130-052-301). CD45^+^ cells were positively selected using QuadroMACS separator LS columns (Miltenyi Biotec, #130-091-051, #130-042-401), according to the manufacturer’s protocol. The sorted cells were stained with CD45 (BD, #5562848), and 7-AAD (BD, #344563) antibodies and analyzed by flow cytometry to confirm successful selection.

### 2.9 RNA sequencing

RNA was isolated using TRIzol (Thermo Fisher Scientific), and total RNA integrity number (RIN) was assessed using TapeStation RNA screentape (Agilent, #5067-5576). Only high-quality RNA samples with a RIN greater than 7.0 were used for library preparation. The cDNA libraries were prepared using the Illumina TruSeq Stranded mRNA Sample Prep Kit (Illumina, Inc., San Diego, CA, USA, #RS-122-2101). Indexed libraries were subsequently sequenced on an Illumina NovaSeq platform (Illumina, Inc., San Diego, CA, USA) using paired-end (2×100 bp) sequencing. After quality filtering with Trimmomatic (v0.38), raw sequencing data were aligned to the reference genome (mm10) using HISAT2 (v2.1.0) (19), and the sequence reads were counted with StringTie (v2.1.3) (20). Differential gene expression was statistically determined using DESeq2 (v1.38.3) (21). Gene ontology analysis was performed using clusterProfiler (v4.14.4) (22). All data analysis and visualization of differentially expressed genes was conducted using R (version 4.4.1). The data have been deposited in NCBI’s Gene Expression Omnibus and are accessible through GEO Series accession number GSE287710 (https://www.ncbi.nlm.nih.gov/geo/query/acc.cgi?acc=GSE287710).

### 2.10 Analysis of TCGA data

TCGA-SARC dataset was accessed using the TCGAbiolinks package (v2.34.0) (23). Survival analysis was conducted using the survival R package (v3.8-3), and Kaplan-Meier plots were visualized using the survminer R package (v0.5.0).

### 2.11 Sample size and replicates

All *in vitro* experiments were performed with at least three biological replicates (independent experiments) and at least three technical replicates per condition, unless otherwise specified.

## 3 Result

### 3.1 Doxorubicin treatment induces upregulation of inflammatory cytokines via cytoplasmic DNA leakage

**Figure 1:**
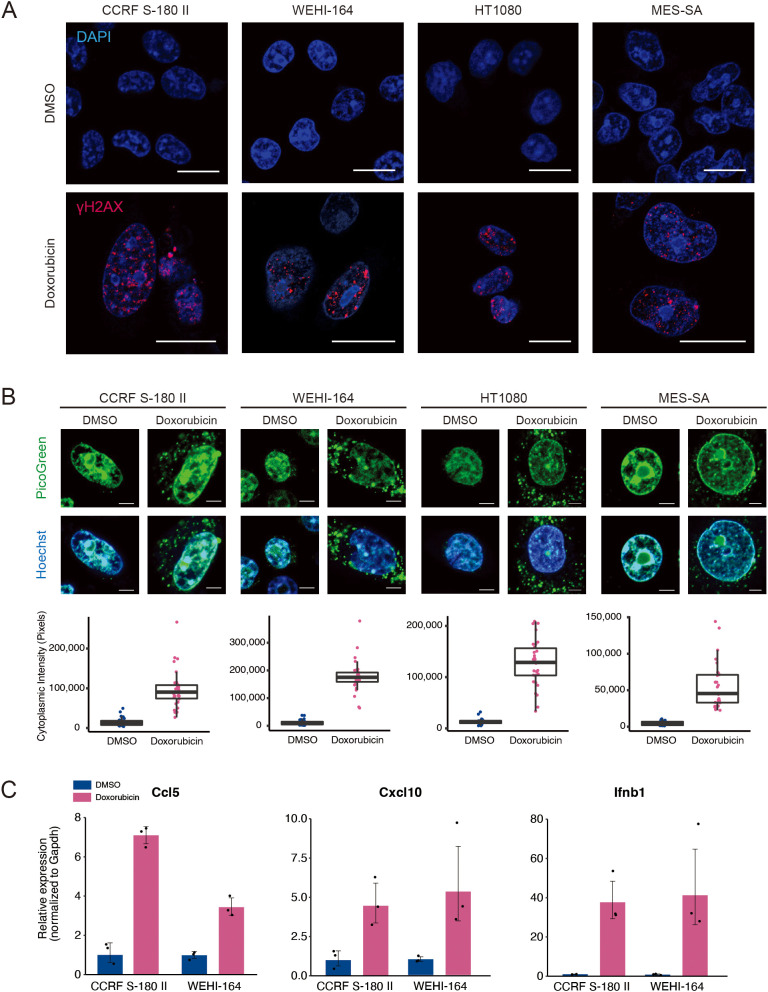
Doxorubicin treatment leads to the upregulation of proinflammatory cytokines. **(A)** Immunofluorescence staining of γH2AX after doxorubicin treatment of mouse (CCRF S-180 II and WEHI-164) and human (HT1080 and MES-SA) soft-tissue sarcoma (STS) cell lines. Scale bars, 20 μm. **(B)** PicoGreen staining and quantification after treatment of STS cell lines with doxorubicin. Scale bars, 5 μm. **(C)** RT-qPCR analysis for *Ccl5*, *Cxcl10*, and *Ifnb1* expression in mouse STS cells treated with dimethyl sulfoxide (DMSO) or doxorubicin at their respective IC_50_ doses for 48 hours. All experiments were performed in at least three biological replicates and repeated independently at least three times.

Cytotoxic agents, such as cisplatin and etoposide, induce the production of inflammatory cytokines via a STING-dependent mechanism (24). To investigate whether doxorubicin exerts a similar effect, we assessed its ability to induce type I IFN signature genes in STS cell lines. Mouse (CCRF S-180 II and WEHI-164), and human (HT1080 and MES-SA) STS cell lines were treated with doxorubicin at their respective IC_50_ concentrations (**Supplementary Figure 1**). Double-stranded DNA breaks (DSBs) were prominently detected via γH2AX staining (**Figure 1A**). DSBs can lead to cytoplasmic DNA leakage, which subsequently activates the STING pathway (25). Consistent with this, doxorubicin treatment caused a significant increase in cytoplasmic DNA levels in STS cells (**Figure 1B**), resulting in the upregulation of STING at the protein level and increased expression of type I IFN signature genes, including *Ccl5*, *Cxcl10*, and *Ifnb1* in mouse sarcoma cells, and CCL5, CXCL10, IFNB1 in human sarcoma cells (**Figure 1C**, and **Supplementary Figures 1B, C**). Collectively, these findings indicated that doxorubicin induces the activation of STING pathway via DSB-mediated DNA leakage into the cytoplasm, leading to the upregulation of type I IFN signature genes.

### 3.2 Knockout of *Sting1* abrogates doxorubicin-mediated upregulation of inflammatory cytokines

**Figure 2:**
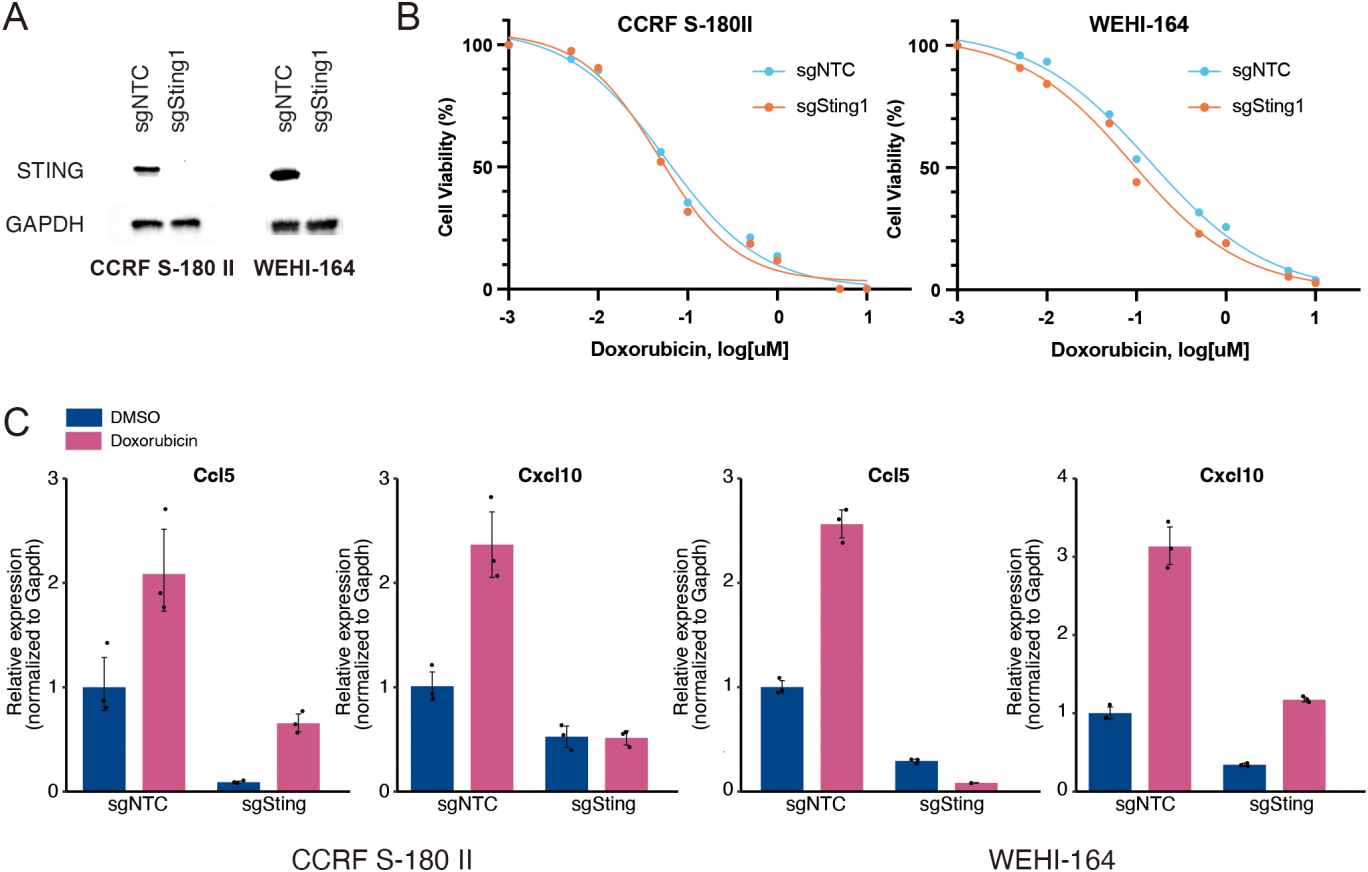
Knockout of *Sting1* attenuates doxorubicin-induced upregulation of proinflammatory cytokines. **(A)** Western blotting for STING expression in mouse soft-tissue sarcoma (STS) cells (CCRF S-180 II and WEHI-164) with *Sting1* deletion (sgSting1) and control (sgNTC). **(B)** Dose-response curves for mouse STS cells with sgSting1 and sgNTC treated with doxorubicin. **(C)** RT-qPCR analysis for *Ccl5* and *Cxcl10* expression in mouse STS cells with sgSting1 and sgNTC. All experiments were performed in at least three biological replicates and repeated independently at least three times.

To confirm whether the doxorubicin-induced expression of type I IFN signature genes is mediated by the activation of STING pathway, we knocked out *Sting1* in two mouse STS cell lines using the CRISPR-Cas9 method (**Figure 2A**). The proliferation rates of *Sting1*-knockout (*Sting1*-KO) cell lines were comparable to those of non-target controls, and their responses to doxorubicin were also similar *in vitro* (**Figure 2B** and **Supplementary Figure 2**). However, while the doxorubicin-induced upregulation of *Ccl5* and *Cxcl10* was observed in both non-target controls and Sting1-KO cells, their relative expression levels remained significantly attenuated in both *Sting1*-KO cell lines (**Figure 2C**). These findings corroborate the fact that the activation of STING pathway mediates doxorubicin-induced upregulation of type I IFN signaling. Furthermore, because STING deficiency did not affect the antitumor effect of doxorubicin *in vitro* where immune cells are absent, we sought to investigate the role of STING pathway in mediating the antitumor effect of doxorubicin in an immunocompetent *in vivo* model.

### 3.3 Addition of STING agonist potentiates the antitumor effect of doxorubicin

**Figure 3:**
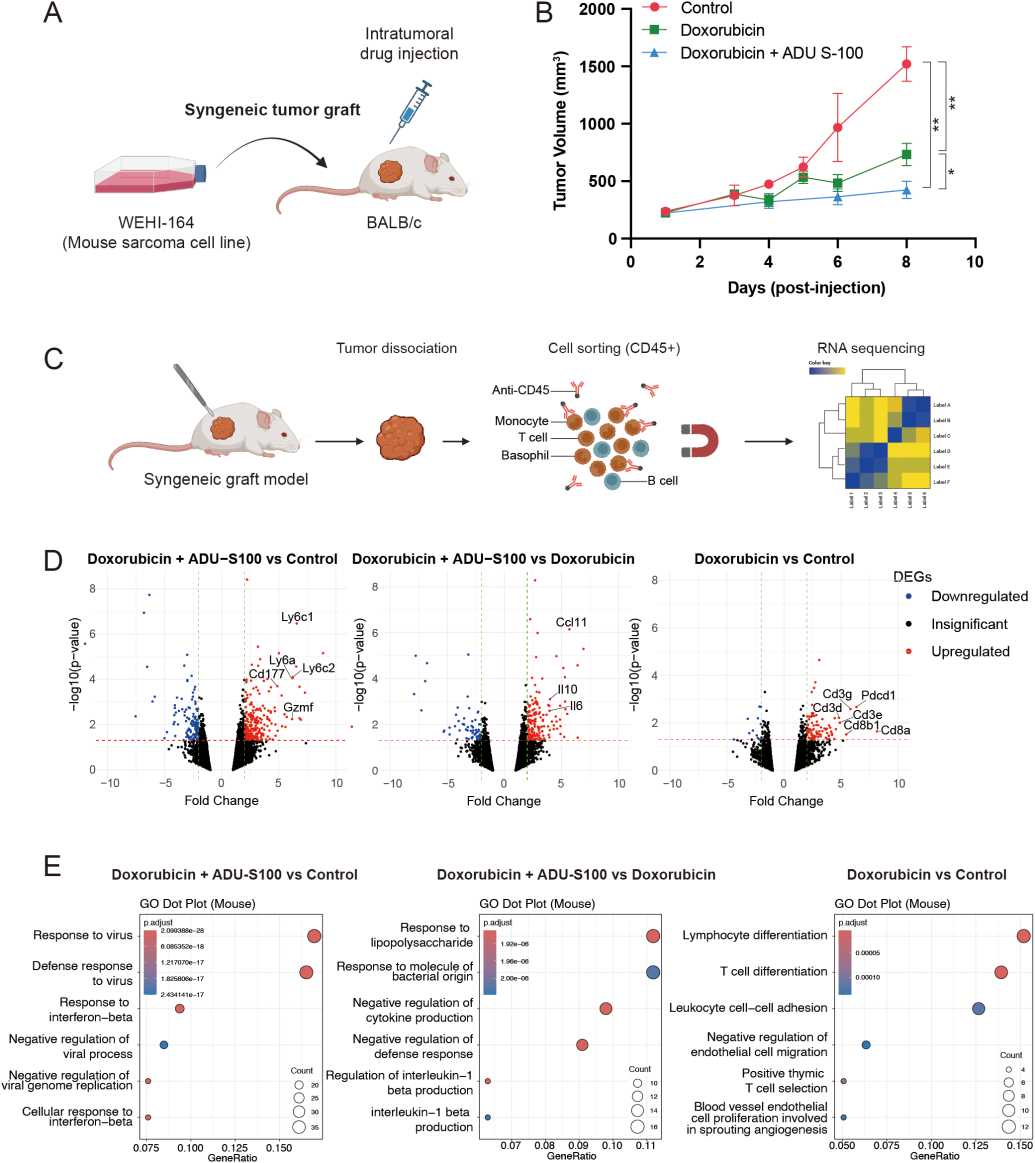
STING agonist ADU-S100 enhances the antitumor effect of doxorubicin in a syngeneic graft model of soft-tissue sarcoma (STS). **(A)** Schematic representation of the generation of syngeneic graft model and drug treatment *in vivo*. **(B)** Tumor sizes in grafted BALB/c mice (*n* = 5 per group) treated with control vehicle, doxorubicin alone, or doxorubicin combined with ADU-S100. *P < 0.05, **P < 0.01. **(C)** Schematic representation of tumor dissociation and immunomagnetic sorting of CD45^+^ cells for RNA sequencing. **(D)** Volcano plots showing differentially expressed genes (DEGs) in CD45^+^ cells, comparing (left) doxorubicin + ADU-S100 versus control, (middle) doxorubicin + ADU-S100 versus doxorubicin alone, and (right) doxorubicin versus control. **(E)** Gene ontology analysis of DEGs, comparing (left) doxorubicin + ADU-S100 versus control, (middle) doxorubicin + ADU-S100 versus doxorubicin alone, and (right) doxorubicin versus control. RNA sequencing was performed using biological triplicates for each treatment group.

Type 1 IFN signaling promotes proinflammatory responses, which is critical for mediating the antitumor effect of doxorubicin *in vivo* (11). Therefore, we investigated whether activating the STING pathway would potentiate the antitumor effects of doxorubicin using an *in vivo* syngeneic model, wherein WEHI-164 cells were grafted into BALB/c mice. For inducing the activation of STING pathway, we used ADU-S100, a STING agonist that has been tested in clinical trials. When the grafted tumors reached a size of 200 mm^3^, doxorubicin was administered either alone or in combination with ADU-S100 (**Figure 3A**). Intratumoral injection of doxorubicin alone suppressed the growth of the grafted tumor. However, its antitumor effect was significantly enhanced by coadministration of ADU-S100 (**Figure 3B**). To determine whether this enhancement was primarily due to ADU-S100 itself, we repeated the experiment including an additional group treated with ADU-S100 alone. Tumor volumes in the combination group were smaller than those in either monotherapy group, corroborating that STING pathway activation by ADU-S100 enhances the antitumor efficacy of doxorubicin *in vivo* (**Supplementary Figure 3**).

### 3.4 RNA-sequencing of tumor-infiltrating leukocytes revealed upregulation of innate immune pathway in tumors treated with the STING agonist

Given that activation of the STING pathway induces proinflammatory responses, we sought to identify which type of immune cell signaling within the tumor microenvironment (TME) contributes to the observed antitumor effects. To address this, we harvested tumors treated with the control vehicle, doxorubicin alone, or the doxorubicin and ADU-S100 combination. The tumors were dissociated, and immune cells within the TME were isolated using CD45-linked microbeads and magnetic-activated cell sorting. RNA sequencing was subsequently performed on the CD45^+^ cell population (**Figure 3C**). In the analysis of differentially expressed genes (DEGs), doxorubicin treatment alone led to the upregulation of *Cd3*, *Cd8*, and *Pdcd1* in the immune cells within the TME (**Figure 3D**, right panel). Gene ontology (GO) analysis of the DEGs further supported this finding, showing enrichment of T-cell signaling pathways (**Figure 3E**, right panel). When comparing the doxorubicin and ADU-S100 combination with either doxorubicin alone or the control vehicle, significant upregulation of genes associated with monocyte/macrophage lineages (*Ly6a*, *Ly6c1*, and *Ly6c2*), and of cytokines or chemokines (*Ccl11*, *Il10*, and *Il6*) that promote immune cell infiltration into the TME was noted (**Figure 3D**, left and middle panel). GO analysis further confirmed these findings, showing enrichment of innate immune signatures and activation of proinflammatory responses (**Figure 3E**, left and middle panel).

### 3.5 Upregulated genes in STING agonist-treated tumors are correlated with improved survival in TCGA-SARC cohort

**Figure 4:**
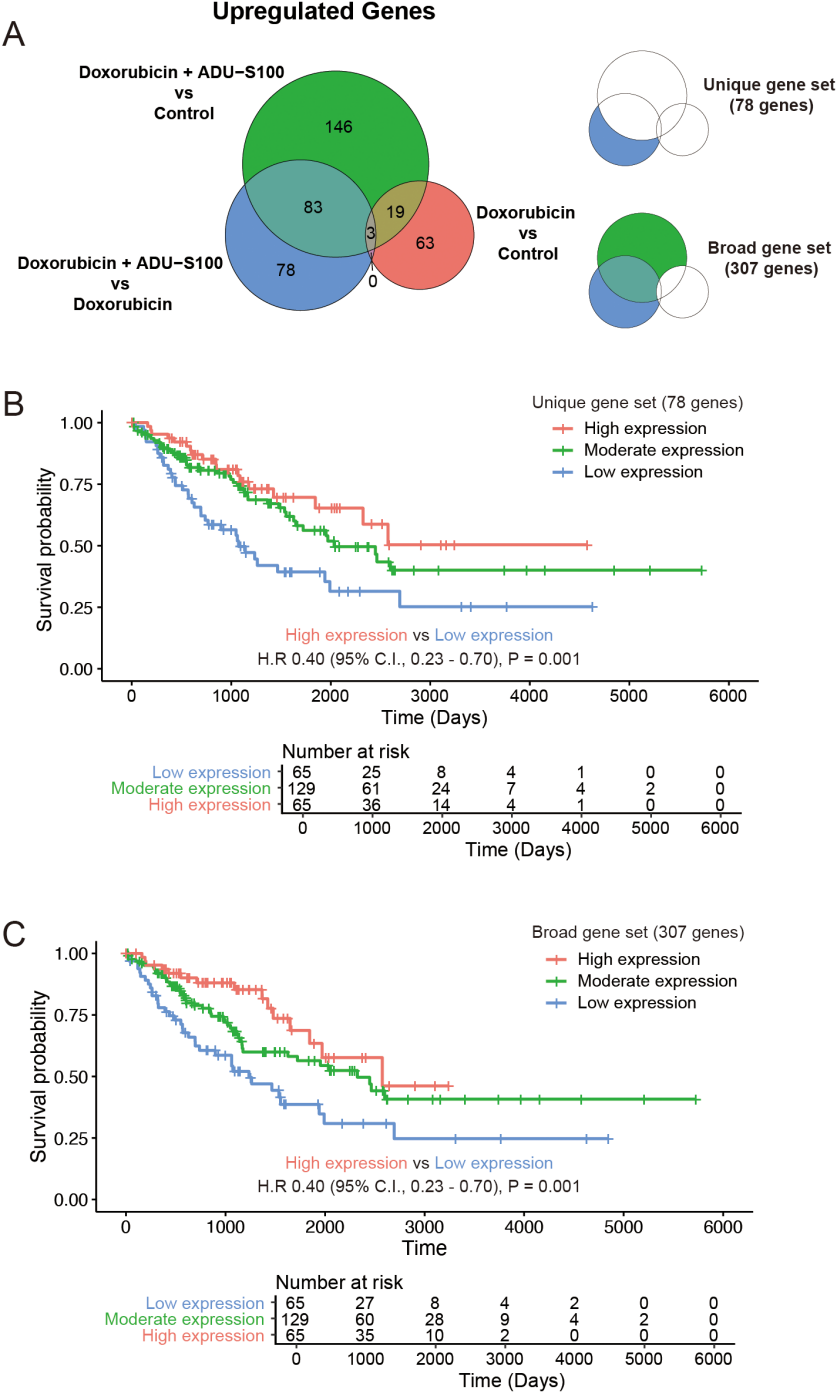
Genes upregulated in STING agonist-treated tumors are correlated with improved survival in human soft-tissue sarcoma (STS). **(A)** Venn diagram showing differentially upregulated genes. **(B)** Kaplan–Meier plot of the overall survival in the Cancer Genome Atlas Sarcoma (TCGA-SARC) cohort stratified by the expression status of the “unique gene set.” **(C)** Kaplan–Meier plot of the overall survival in TCGA-SARC cohort stratified by the expression status of the “broad gene set.”.

Comparisons of the upregulated genes in immune cells from tumors treated with doxorubicin and ADU-S100 with those treated with doxorubicin alone or the control vehicle reflected gene signatures consistent with the activation of STING pathway. We subclassified the DEGs that were exclusively upregulated (fold change >2 and raw *p*-value <0.05) in the doxorubicin plus ADU-S100 group compared with that in the doxorubicin-alone group as “unique gene set (*N* = 78).” Additionally, genes upregulated in the doxorubicin plus ADU-S100 group compared with that in the control vehicle group, but not overlapping with the upregulated DEGs in the doxorubicin versus control comparison, were combined with the “unique gene set” and collectively termed the “broad gene set (*N* = 307)” (**Figure 4A**, and **Supplementary Tables 1, 2**). We sought to explore whether the gene sets reflecting the activation of STING pathway in the TME of our experimental model could predict the clinical outcomes in patients with STS. Using transcriptome data of The Cancer Genome Atlas Sarcoma (TCGA-SARC), we performed single-sample gene set enrichment analysis (ssGSEA) with the “unique gene set,” and the “broad gene set.” We then compared the overall survival among groups with high (top 25^th^ percentile), moderate (25^th^–75^th^ percentile), and low (bottom 25^th^ percentile) expression levels. Patients with high expression levels of either the “unique gene set” or the “broad gene set” had significantly longer overall survival compared with those with low expression levels (**Figures 4B, C**). These findings indicated that patients with STS exhibiting higher expression of STING activation markers have improved clinical outcome, supporting our experimental model and highlighting the potential of strategies aimed at activating the STING pathway for STS therapy.

## 4 Discussion

Doxorubicin is a chemotherapeutic agent widely used for the treatment of various malignancies; however, the precise mechanisms for the antitumor effect of doxorubicin remain incompletely understood (26). Although the ability of doxorubicin to induce DNA damage is the most well-characterized and critical mode of its action, recent studies have highlighted its role in modulating the TME and triggering T-cell responses (27, 28). Furthermore, evidence suggests that antitumor immunity is essential for the therapeutic efficacy of doxorubicin. Mattarollo et al. demonstrated that CD8^+^ T cells and IFN-γ production are necessary for the therapeutic effects of doxorubicin (10). Additionally, Sistigu et al. demonstrated that a type I IFN signature is essential for the therapeutic efficacy of doxorubicin and that this signature could also predict clinical responses to anthracycline-based chemotherapy in various cohorts of patients with breast cancer (11).

STS is generally considered immunologically “cold,” and clinical trials of immune checkpoint inhibitors have yielded unsatisfactory outcomes (29). Combinations of doxorubicin with the anti-PD1 antibody pembrolizumab have also been evaluated in clinical trials but have not demonstrated significant improvements in the overall survival (30, 31). The limited efficacy of doxorubicin and anti-PD1 blockade may be attributed to the absence of robust antitumor immune responses within the TME. As this represents a significant challenge for many solid tumors, strategies aimed at converting “cold” tumors into “hot” tumors have come under extensive research focus (32). Given these unmet clinical needs, our model of combining a STING agonist with doxorubicin offers a novel therapeutic approach for STS.

Using our experimental model, we demonstrated that the doxorubicin-induced upregulation of proinflammatory cytokines is mediated via activation of the STING pathway, which is triggered by cytoplasmic DNA leakage resulting from DSBs. Furthermore, using a syngeneic graft model of STS, we showed that enhancing this pathway by combining the STING agonist ADU-S100 with doxorubicin could potentiate the antitumor activity via the upregulation of innate immune response signaling.

ADU-S100, a synthetic cyclic dinucleotide that activates the STING pathway, has been evaluated in clinical trials. During the course of our research, results from two phase 1 trials were published: one investigating ADU-S100 as a monotherapy in a dose-escalation study and the other examining the efficacy of its combination with spartalizumab, an anti-PD1 antibody (16, 17). Both studies demonstrated that ADU-S100 was well tolerated, but the clinical efficacy was limited both as a monotherapy and when used in combination with anti-PD1 therapy. However, novel STING agonists are under active investigation in numerous ongoing clinical trials (33). Additionally, in contrast to cyclic dinucleotides, which are primarily administered via an intratumoral injection, small molecules that directly bind to STING and enable systemic administration have been characterized (34, 35). Innovative approaches, such as antibody-drug conjugates of STING agonists and targeted protein upregulation of STING, have been also reported and are expected to be evaluated in clinical studies (36, 37).

Our study had several limitations. First, our model relied on a single syngeneic graft model using a murine STS cell line. However, in our in silico analysis of TCGA-SARC cohort with the genes upregulated in STING agonist-treated tumors, patients with higher expression of genes upregulated in STING agonist-treated tumors showed improved overall survival. This finding supports the notion that enhancing STING signaling could potentially lead to improved clinical outcomes in human STS. Second, while we utilized intratumoral injection to ensure localized delivery of doxorubicin to the tumor microenvironment and minimize systemic exposure, this route is not routinely used in clinical practice. We did not evaluate alternative routes such as intravenous or intraperitoneal administration, which would be essential for future translational studies assessing the clinical feasibility of this therapeutic strategy. Third, we did not include a monotherapy group receiving ADU-S100 alone. The absence of this group precludes definitive conclusions regarding the additive or synergistic nature of the combination treatment. While our study focused on evaluating the combinatorial effects, we acknowledge that without the ADU-S100-only group, the individual contribution of STING activation remains uncertain. These issues should be addressed in future studies to refine the therapeutic relevance of this approach. Fourth, the STING agonist ADU-S100, used in our experiments, has shown limited efficacy in clinical trials, raising questions about the feasibility of combining this class of drugs for human patients with STS. Nonetheless, given recent advancements in strategies to enhance STING activity, our study provides proof-of-concept data to justify exploring this combination in future clinical trials.

In conclusion, we demonstrate that the activation of STING pathway enhances the antitumor efficacy of doxorubicin in STS. Given the limited advancements in systemic treatment of STS, including targeted therapies and immune checkpoint inhibitors, incorporating the activation STING pathway into standard chemotherapy regimens may offer a promising strategy to improve clinical outcomes for this challenging disease.

## Data Availability

The datasets presented in this study can be found in online repositories. The names of the repository/repositories and accession number(s) can be found below: https://www.ncbi.nlm.nih.gov/geo/query/acc.cgi?acc=GSE287710, GSE287710.
