# Supplementary material for: Activation of the STING pathway potentiates the antitumor efficacy of doxorubicin in soft-tissue sarcoma
Source: Front Oncol. 2025 Dec 12;15:1634503. doi: 10.3389/fonc.2025.1634503 (PMC12740912; doi:10.3389/fonc.2025.1634503)
Supplement: Supplementary file 1 [file DataSheet1.docx]

**Supplementary Figure 1**

**
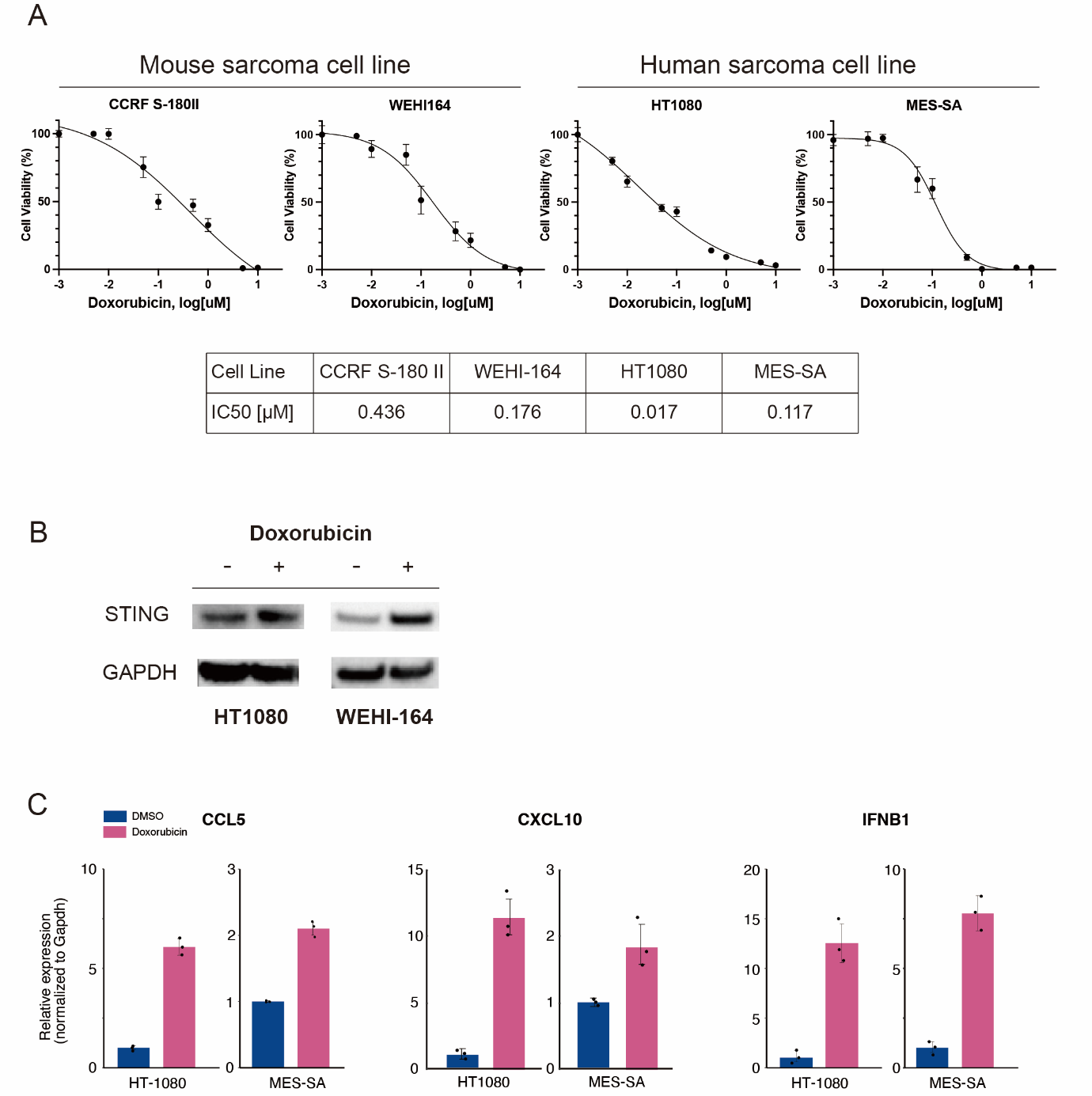
**

(A) Dose response curves and IC50 values of mouse and human sarcoma cell lines to doxorubicin.

(B) Western blot result for STING and GAPDH in HT-1080 (human) and WEHI-164 (mouse) sarcoma cell lines.

(C) RT-qPCR analysis for CCL5, CXCL10, and IFNB1 expression in human STS cells (HT-1080 and MES-SA) treated with dimethyl sulfoxide (DMSO) or doxorubicin at their respective IC_50_ doses for 48 hours.

All experiments were performed in at least three biological replicates and repeated independently at least three times.

**Supplementary Figure 2**


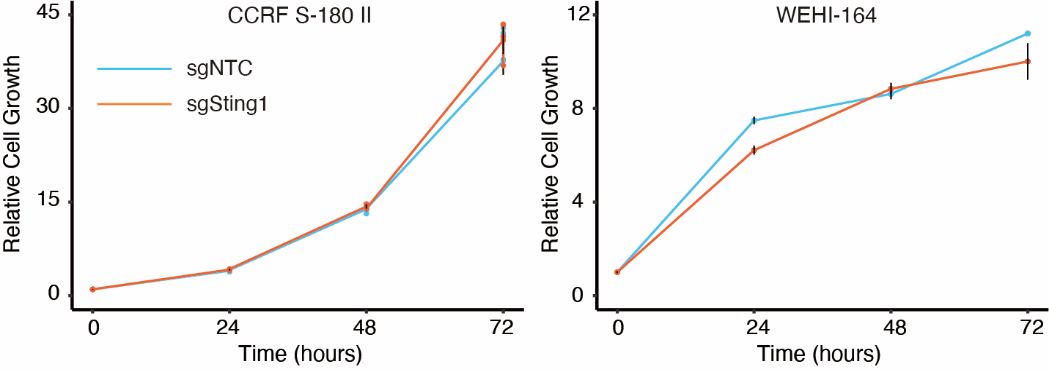


Cell proliferation growth curves of mouse STS cells with sgSting1, and sgNTC.

**Supplementary Figure 3**


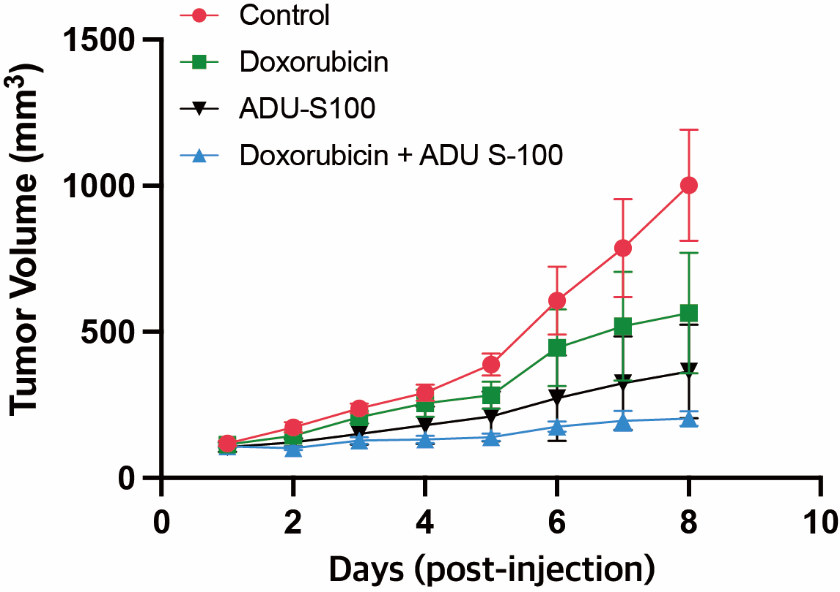


Tumor sizes in grafted BALB/c mice (*n* = 5 per group) treated with control vehicle, doxorubicin alone, ADU-S100 alone, or doxorubicin combined with ADU-S100.
