# Supplementary material for: Activation of the STING pathway potentiates the antitumor efficacy of doxorubicin in soft-tissue sarcoma
Source: Front Oncol. 2025 Dec 12;15:1634503. doi: 10.3389/fonc.2025.1634503 (PMC12740912; doi:10.3389/fonc.2025.1634503)
Supplement: Supplementary file 2 [file Table1.docx]

| **Supplementary Table 1. List of genes in the ‘Unique gene set’** | | | | | |
| --- | --- | --- | --- | --- | --- |
| Prdm1 | Serping1 | Cd80 | Chga | Cyp1b1 | Egr1 |
| Eln | Fosb | Fosl2 | Gem | Gjb5 | Cxcl1 |
| Has1 | Ifrd1 | Igf1 | Il1a | Irf4 | Mmp3 |
| mt-Rnr1 | mt-Rnr2 | TrnA | TrnL1 | TrnP | TrnT |
| TrnW | Mylpf | Nr4a2 | Pde4b | Ptx3 | Rel |
| Cxcl12 | Serpina1b | Tagln | Tnfaip2 | Slc7a11 | Arrdc4 |
| Ms4a4d | Grtp1 | Tmem88 | Coq10b | Cavin4 | Lipn |
| Gprc5c | Medag | Lrrc63 | Enkur | Amdhd1 | Dpep3 |
| Zfp429 | Cfap126 | Arl5b | Mir17hg | Il33 | Cadm3 |
| AI606473 | Osgin2 | Trim30d | Nrg1 | Nlrp3 | Kdm6b |
| Alpk2 | Tnfaip8l3 | Chd7 | Tnfsf15 | Tmem26 | Zfp667 |
| Ugt1a1 | Rplp2-ps1 | Gm8750 | Gm8752 | Gm15708 | Gm30732 |
| Gm32249 | Gm33272 | Gm38504 | Gm34643 | Gm38794 | Gm4169 |

| **Supplementary Table 2. List of genes in the ‘Broad gene set’** | | | | | | | | | |
| --- | --- | --- | --- | --- | --- | --- | --- | --- | --- |
| Adora2a | Adora2b | | Apoc1 | | Apod | | Areg | | Arg2 |
| Bcl2l11 | Prdm1 | | Serping1 | | Car3 | | Cd80 | | Chga |
| Chl1 | Socs1 | | Crem | | Cyp1b1 | | Cyp7b1 | | Des |
| Dusp2 | S1pr3 | | Egr1 | | Eln | | Fmo1 | | Fosb |
| Fosl2 | Gem | | Gjb5 | | Cxcl1 | | Gstt1 | | Has1 |
| Id3 | Irgm1 | | Ifrd1 | | Igf1 | | Il10 | | Il1a |
| Il6 | Inhba | | Irf1 | | Irf4 | | Mxd1 | | Maff |
| Mmp3 | mt-Rnr1 | | mt-Rnr2 | | TrnA | | TrnL1 | | TrnP |
| TrnT | TrnW | | Mx2 | | Gadd45b | | Mylpf | | Nfkbie |
| Nr4a3 | Nos2 | | Nr4a2 | | Pde4b | | Procr | | Ptger1 |
| Ptx3 | Rel | | Ccl11 | | Cxcl12 | | Slfn4 | | Serpina1b |
| Spic | Tagln | | Timp1 | | Tnfaip2 | | Tnfaip6 | | Traf1 |
| Wnt11 | Oas1g | | Slc7a11 | | Sertad1 | | Ramp3 | | Grasp |
| Rrad | Gpr132 | | Ly6i | | Dpys | | Arrdc4 | | Ms4a4d |
| Grtp1 | Tmem88 | | Spats2l | | Coq10b | | Cavin4 | | 1110002J07Rik |
| Lipn | Gprc5c | | Medag | | Lrrc63 | | Enkur | | Flrt3 |
| Rarres2 | Amdhd1 | | Dpep3 | | Zfp429 | | Dmkn | | Errfi1 |
| Cfap126 | Rab11fip1 | | Arl5b | | Mir17hg | | Il33 | | Cadm3 |
| AI606473 | Ildr1 | | B3gnt5 | | Osgin2 | | Trim30d | | Nrg1 |
| Nlrp3 | Kdm6b | | Ccno | | Alpk2 | | Cytip | | Oasl1 |
| Spon1 | Prss50 | | Dusp5 | | Tnfaip8l3 | | Nod2 | | Slc4a11 |
| Ceacam19 | Chd7 | | Tnfsf15 | | Tmem26 | | Xaf1 | | Zfp667 |
| Ugt1a1 | Tnip3 | | Gm7582 | | Gm7609 | | Rplp2-ps1 | | Gm8221 |
| Gm8750 | Gm8752 | | Ifi208 | | Isg15 | | Mir22hg | | Gm18445 |
| Cbarp | Gm15056 | | Gm15708 | | Gm15987 | | Gm30732 | | Gm32249 |
| Gm33272 | Gm38504 | | Gm34643 | | Gm38510 | | Ptgs2os2 | | Gm36753 |
| Gm38794 | Gm41348 | | Gm41699 | | Gm42049 | | LOC108167347 | | Afp |
| Cd5l | Ass1 | | Fabp7 | | Car4 | | Casp4 | | Cd7 |
| Chil3 | Clu | | Cryba4 | | Gadd45a | | F10 | | Flt1 |
| Gbp2 | Gzmd | | H2-Aa | | Cfb | | H2-Eb1 | | H2-T10 |
| H2-T22 | H2-T23 | | Hp | | Id1 | | Cxcl10 | | Ifi204 |
| Ifi47 | Ifit1 | | Ifit3 | | Ifnb1 | | Il18bp | | Igtp |
| Il1b | Il1r2 | | Itgb7 | | Kcnab1 | | Kcnq1 | | Napsa |
| Psmb9 | Ltb4r1 | | Pgf | | Pou3f1 | | Psme2 | | S100a6 |
| Saa3 | Ccl5 | | Ccl8 | | St8sia1 | | Slfn2 | | Slpi |
| Sdc1 | Cd40 | | Trex1 | | Cmpk2 | | Oasl2 | | Slco2a1 |
| Map2k3os | Usp18 | | Klrk1 | | C1s1 | | Irf7 | | Mtmr7 |
| Irgm2 | Mefv | | Sfn | | Cxcl11 | | Pkp3 | | Stap1 |
| Isg20 | Rsad2 | | Zbp1 | | Zbtb32 | | Ms4a4b | | Ms4a4c |
| Rgcc | Tespa1 | | Rtp4 | | Tppp3 | | Ifitm1 | | Pdgfrl |
| Bst2 | Rhou | | Ifi35 | | Apol9b | | Cnn3 | | 1600014C23Rik |
| LOC73899 | Stx11 | | 4930459C07Rik | | Slamf7 | | Ankrd55 | | Clec2d |
| AI839979 | Hepacam2 | | Cdc42ep2 | | AW112010 | | Ssc4d | | Syt17 |
| Ifit1bl2 | Cd209a | | Fam83f | | Phf11d | | Rnd1 | | Ifi205 |
| Gpr55 | Tmeff1 | | Samd11 | | Gpat3 | | Kctd14 | | Yjefn3 |
| Gm4963 | Tarm1 | | Oas3 | | Stfa2l1 | | Gimap9 | | Igf2bp2 |
| B930059L03Rik | Enpp6 | | 1700006J14Rik | | A530064D06Rik | | Il1bos | | Ankdd1a |
| Egfl7 | A530032D15Rik | | 6430531B16Rik | | Glipr2 | | Ndufa4l2 | | Cstdc4 |
| Trim30c | Ifi214 | | Gramd2 | | Gm12185 | | BC147527 | | Gm12250 |
| Ifit3b | Ifit1bl1 | | Gm9733 | | Gm14010 | | A630012P03Rik | | LOC101055758 |
| Gm12589 | Ifi206 | | Gm36856 | | Gm38922 | | Gm40491 | | Gm13822 |
| LOC115490433 | |  | |  | |  | |  | |
